# Genetic Diversity and Population Structure of Five Pig Breeds from Chongqing, China

**DOI:** 10.3390/ani15172610

**Published:** 2025-09-05

**Authors:** Xi Long, Lidan Zhang, Yu Pan, Liang Zhang, Zhi Tu, Lijuan Zhang, Qing Wang, Hongmei Pan, Zongyi Guo

**Affiliations:** 1 Chongqing Academy of Animal Science, Chongqing 402460, China; lx13618288075@outlook.com (X.L.); panyu@cqaa.cn (Y.P.); zhangl@cqaa.cn (L.Z.); tuz@cqaa.cn (Z.T.); zhanglj@cqaa.cn (L.Z.); 2 National Center of Technology Innovation for Pigs, Chongqing 402460, China; gelp@cqaa.cn (L.Z.); qingwang_cq@163.com (Q.W.)

**Keywords:** domestic pig breeds, population structure, genetic diversity, conservation

## Abstract

Five pig breeds have been developed in Chongqing and are economically important livestock resources in this region: Hechuan Black pigs (HC), Luopanshan pigs (LP), Penzhou Mountain pigs (PZ), Quxi pigs (QX), and Rongchang pigs (RC). Understanding the genetic diversity and population structure of pig breeds can help improve the conservation and management of their genetic resources. This study sequenced SNPs in 188 pigs from five breeds in Chongqing, China, using a 50K SNP chip. Of the 45,211 detected SNPs, 9442 were excluded after quality control, and 35,769 were included in the analysis. Among the five breeds, QX had the highest genetic diversity. Principal component analysis and phylogenetic trees showed that the five populations were phylogenetically distinct. Population structure analysis identified five subpopulations at K = 5, indicating that RC were genetically independent. RC had the fastest linkage disequilibrium (LD) decay, suggesting weaker selection pressure. In turn, HC exhibited the slowest decay, indicating stronger selection pressure and closer relatedness within this population. The analysis of genetic differentiation indices revealed that LP and HC had the highest degree of differentiation (0.1958), while QX and PZ had the lowest (0.0748). In conclusion, we found differences in population structure among breeds, providing a basis for conserving genetic diversity and improving pig breeds.

## Simple Summary

Chongqing, a municipality directly under the Central Government of China, is located in the southwest of the country and has a subtropical monsoon climate. Five pig breeds with distinct genetic characteristics evolved in this region due to climate, topography, and human activities. These breeds have high-quality meat and are resistant to heat, humidity, and disease. This study used SNP chips to investigate the genetic diversity and population structure of these pig breeds to help conserve livestock diversity and genetic resources, thereby increasing the genetic diversity of pig breeds worldwide.

## 1. Introduction

As one of the earliest domesticated animals, pigs have provided humans with a stable source of meat and fertilizer, promoting the development of agriculture. China is one of the important origins of pig domestication, with a history of pig farming spanning thousands of years [1,2]. Given China’s vast territory, pig breeds in various regions have adapted well to local conditions through natural selection and breeding. For instance, Min pigs are entirely covered with black fur, which improves their survival during winter [3]. Hainan pigs are relatively small and have short and sparse fur, which improves their adaptation to hot and humid environments [4,5]. Tibetan pigs have well-developed hearts and lungs, which improve adaptation to the high altitudes and low oxygen levels of the Qinghai–Tibet Plateau [6,7]. Chinese pig breeds have superior reproductive performance, high-quality meat, and high stress resistance, enriching their gene pools worldwide [8,9,10,11,12]. These traits improve pig breeds and help create new ones. In the 18th and early 19th centuries, European countries, including the United Kingdom, imported high-quality Chinese pig breeds, such as Meishan pigs, to improve the reproductive performance of domestic pig populations [13,14].

Although China has many pig breeds with unique genetic characteristics, most of these breeds are fat-type or dual-purpose (meat–fat) breeds, which are characterized by a low percentage of lean meat. In contrast, European breeds such as the White Duroc and Landrace are characterized by short growth cycles and a high percentage of lean meat. Yorkshire, White Duroc, Landrace, and other European breeds were introduced in China to crossbreed with domestic pigs, meeting consumer demands and stimulating the development of new hybrid breeds with higher productivity and market value [15]. However, due to a lack of awareness about the need to protect domestic breeds, many foreign breeds have been used in domestic genetic improvement programs, neglecting the traits and ecological significance of local breeds. Foreign genes spread to local pig populations, causing changes in the gene frequency and genetic structure of these populations [16,17,18,19].

Chongqing, located in the eastern part of the Sichuan Basin in China, has a subtropical monsoon humid climate, characterized by hot and rainy summers and mild dry winters, creating a climate characterized by high temperatures and humidity along with relatively cold weather. Five pig breeds with excellent traits have been developed in Chongqing through selection and breeding: Hechuan Black pigs (HC), Luopanshan pigs (LP), Penzhou Mountain pigs (PZ), Quxi pigs (QX), and Rongchang pigs (RC) [20]. HC, LP, PZ, and QX have medium to large sizes and black fur; nonetheless, each breed has unique features. HC are resistant to high temperatures and high humidity and have a high intramuscular fat content. LP have thick and long fur and tender meat, making it suitable for stewing. PZ are suitable for grazing in mountainous areas and have a strong fat deposition capacity. Their meat is excellent for producing cured meats and ham. QX have short and sturdy limbs, can tolerate coarse feed, and have strong disease resistance. RC have white fur with black spots of varying sizes on the head and around the eyes (hence their popular name, “Panda” pigs). These domestic breeds have superior traits, serving as valuable genetic resources for creating new breeds and supporting the sustainable growth of China’s swine industry. However, the widespread introduction of foreign breeds and the effects of African swine fever epidemics have reduced domestic pig populations and the number of purebred animals, potentially reducing genetic diversity and posing significant challenges for local pig conservation efforts. The latest census shows that QX and LP are on the verge of extinction, while HC are at risk of extinction, underscoring the urgent need for local conservation efforts.

The development of molecular markers and detection technologies has enabled the comprehensive and accurate assessment of genetic diversity and population structure in animal breeds using high-density SNP chips. Previous studies used SNP chips to investigate the genetic diversity of domestic pig breeds, including Fengjing, Tongcheng, Min, and Laiwu pigs [21,22,23,24]. In this study, 50K SNP chips were used to analyze the genetic diversity and population structure of 188 pigs from five pig breeds in Chongqing. The results can help evaluate genetic diversity, the degree of genetic differentiation, and gene exchange among different populations, providing theoretical support for the protection, sustainable development, and utilization of local genetic resources.

## 2. Materials and Methods

### 2.1. Sample Collection

We collected ear tissue samples from 188 pigs (40 HC, 40 LP, 38 PZ, 30 QX, and 40 RC) from conservation and production farms using sterilized ear-piercing pliers. The pig breeds and sample sizes are shown in Table 1. The collected samples were placed in 2 mL cryopreservation tubes containing 75% ethanol and stored at −20 °C for later use.

### 2.2. DNA Extraction

Genomic DNA was extracted using the CWE9600 Magbead Blood DNA Kit (Kangwei Century Biotechnology Co., Ltd., Taizhou, Jiangsu, China), which utilizes magnetic beads. DNA concentration and purity were assessed using the Nanodrop 2000 (Thermo Fisher, Waltham, MA, USA). DNA integrity was evaluated by agarose gel electrophoresis. The qualified DNA samples (OD_260_/OD_280_ = 1.7–2.1 and a concentration ≥ 50 ng/μL) were stored at −20 °C for analysis.

### 2.3. SNP Genotyping and Quality Control

Genomic DNA samples that passed quality control were subjected to SNP genotyping using the CAUPorince 50K platform (Kangpu Sen Agricultural Technology Co., Ltd., Beijing, China). Quality control of SNP loci was performed using PLINK software version 1.90. Based on the following quality control criteria: (1) individual detection rate ≥ 90%, (2) use of autosomal loci, (3) retention of SNP markers with a detection rate ≥ 90%, (4) retention of SNPs with a minimum allele frequency ≥ 0.01, and (5) exclusion of markers with a Hardy–Weinberg equilibrium test *p*-value of less than 10^−6^ [25].

### 2.4. Genetic Diversity Analysis

The genetic diversity of pig breeds was investigated by calculating the minor allele frequency (MAF), expected heterozygosity (He), observed heterozygosity (Ho), effective population size (Ne), proportion of polymorphic marker ratio (PN), and nucleotide diversity (Pi). The MAF, He, Ho, and PN of each population were determined using PLINK version 1.90. Ne was calculated using SNeP version 1.1, and Pi was measured using VCFtools version 0.1.17 [26,27,28]. In addition, raincloud plots of the MAF, Ho, He, and Pi were obtained using R software version 4.2.0.

### 2.5. Analysis of Linkage Disequilibrium (LD)

The degree of LD was evaluated using PopLDdecay version 1.90. The degree of LD was measured using r^2^, which reflects the association between two loci [29].

### 2.6. Genetic Differentiation Index

The genetic differentiation index, FST, measures the genetic similarity between populations. The index ranges from 0 to 1, where 0 indicates random mating between two populations with similar genotypes, and 1 indicates complete isolation with no mating. FST distances were calculated using VCFtools (V0.1.17) software [26].

### 2.7. Principal Component Analysis (PCA)

PCA is a statistical method that reduces dimensionality by transforming a large number of indicators into a few composite indicators (principal components) that retain most of the original information without redundancy. PCA was performed using GCTA version 1.94 [30]. The first two principal components were visualized using R version 4.2.0.

### 2.8. Phylogenetic Tree Analysis

A phylogenetic tree shows the evolutionary relationships and the order of differentiation among populations. Based on the identity-by-state (IBS) genetic distance, the phylogenetic tree was generated using the neighbor-joining method in MegaX version 10.0 [27,31].

### 2.9. Population Structure Analysis

Population structure is the non-random distribution of genetic variations within a species or population. A population can be divided into several subgroups based on geographical distribution and other factors. Members of the same subgroup are more closely related to one another than to members of other subgroups. Population structure analysis improves our understanding of evolutionary processes and can help identify subpopulations by examining the association between genotypes and phenotypes. In this study, various K values were selected based on population data, and the population structure was analyzed using Admixture software version 1.3 [32]. The coefficient of variation (CV) is inversely correlated with the K value, which is used to determine the optimal number of clusters.

### 2.10. TreeMix Analysis

The degree of population differentiation and genetic exchange between pig breeds was evaluated using TreeMix version 1.13 [33]. In the analysis, we designated the RC population as an outgroup and used it as the root of the maximum likelihood tree generated by TreeMix. The optimal number of migration events in the migration model was identified based on the results of the residual analysis.

### 2.11. Genetic Relationships Analysis

The IBS genetic distance matrix was constructed using PLINK version 1.90, and the G matrix was constructed using GCTA version 1.94. We analyzed the kinship among individuals in each population and visualized the results using R version 4.2.0 [27,30].

## 3. Results

### 3.1. Quality Control of SNPs

**Figure 1:**
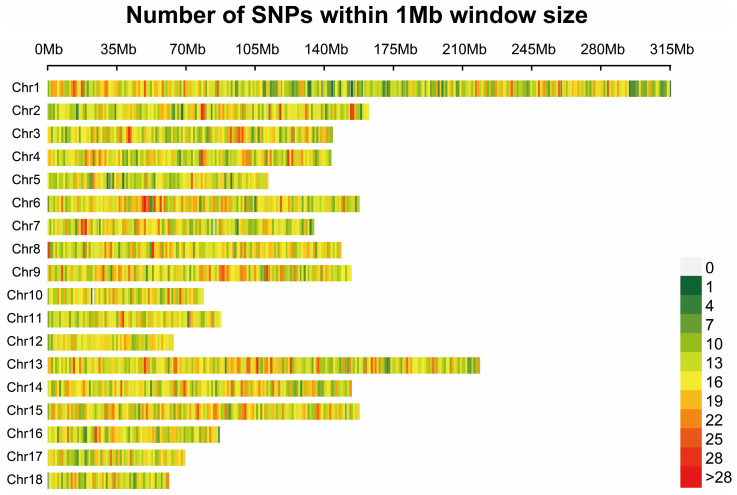
Distribution of single-nucleotide polymorphisms on chromosomes.

More than 90% of the SNP loci were detected in all 188 samples. A total of 45,211 SNPs were identified. After quality control, 35,769 SNPs were analyzed. SNPs were identified on all chromosomes, with the highest number occurring on chromosome 1 (Figure 1).

### 3.2. Analysis of Genetic Diversity

**Figure 2:**
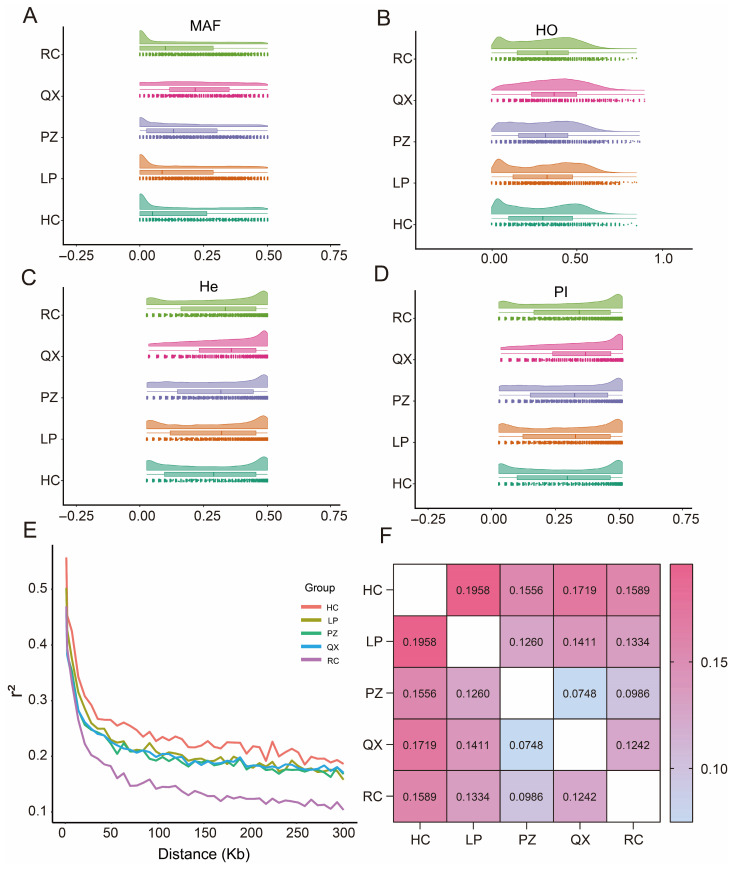
Genetic diversity of five pig breeds from Chongqing, China. (**A**–**D**) Raincloud plots of minor allele frequencies, observed heterozygosity, expected heterozygosity, and nucleotide diversity. (**E**) Linkage disequilibrium (LD) decay plots. The horizontal axis represents the physical distance in kilobases, and the vertical axis represents the average degree of LD (r^2^). (**F**) Genetic differentiation of five pig populations.

The results of MAF, He, Ho, Ne, PN, and Pi are shown in Table 2 and Figure 2. The MAFs of the five pig breeds varied from 0.135 (HC) to 0.229 (QX). The Ho for all populations exceeded the expected heterozygosity. QX had the highest He, Ho, PN, and Pi, at 0.3302, 0.3624, 0.8946, and 0.3359, respectively. In turn, QX had the lowest Ne (2.7). HC had the lowest He, Ho, PN, and Pi values, at 0.2751, 0.2958, 0.5090, and 0.2785, respectively. RC had the highest Ne (4.6). QX had the highest genetic diversity.

The LD decay plot showed that the r^2^ decreased in all five breeds as the distance between the loci increased (Figure 2E). The r^2^ coefficients were highest in HC and lowest in RC. LD decay was highest in RC. The degree of genetic differentiation among pig breeds was determined by calculating FST values (Figure 2F). The genetic differentiation indices between any two populations of LP, PZ, QX, and RC ranged from 0.05 to 0.15, indicating moderate differentiation. The genetic differentiation indices between HC and the other four breeds were above 0.15, indicating high differentiation.

### 3.3. Analysis of the Population Genetic Structure and Phylogenetic Relationships

**Figure 3:**
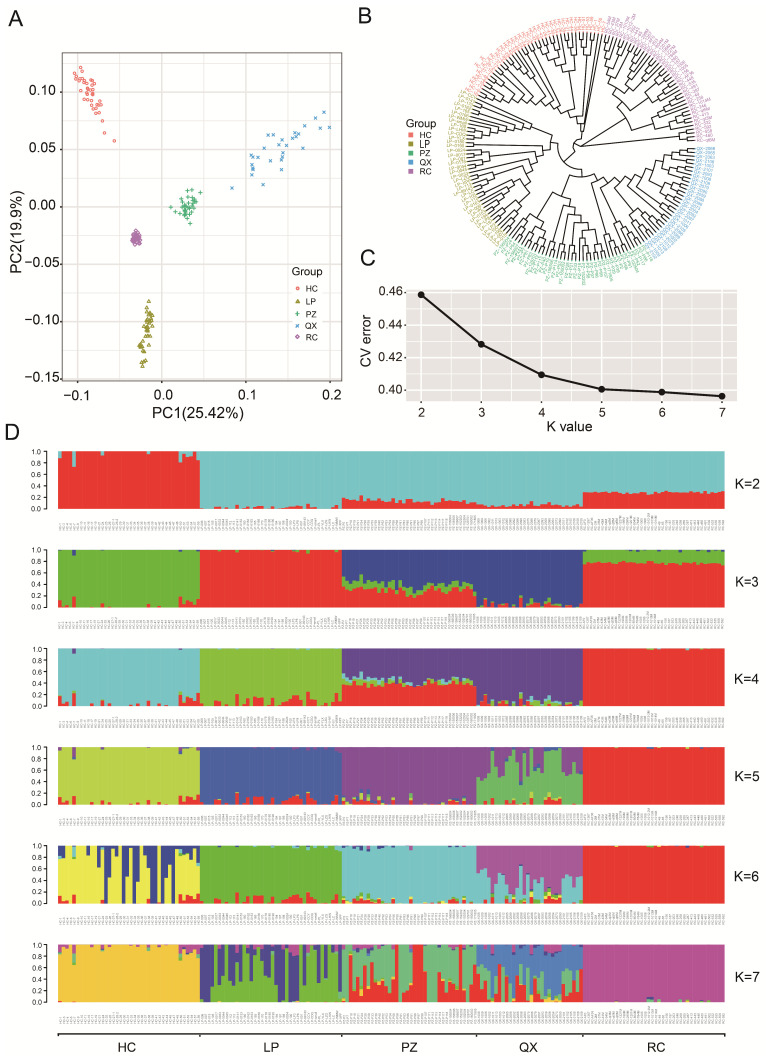
Genetic structures of five pig breeds from Chongqing, China. (**A**) Principal component analysis plots of 188 pigs from five pig breeds. (**B**) Phylogenetic tree of five pig breeds. (**C**) Cross-validation error rate. (**D**) Admixture analysis of ancestral populations (K = 2–7).

PCA results are shown in Figure 3A. The five populations clustered separately, with the first two principal components clearly distinguishing between them, while the RC population was more closely grouped. The phylogenetic tree showed that the RC population formed a distinct branch. In contrast, the HC, LP, PZ, and QX populations formed a branch that separated into four distinct groups (Figure 3B). Multiple K values were analyzed in these populations. CV values are inversely correlated with K values, which are used to determine the optimal number of clusters. The optimal number of clusters was five (Figure 3C). When K was 5, the five populations were genetically independent (Figure 3D).

**Figure 4:**
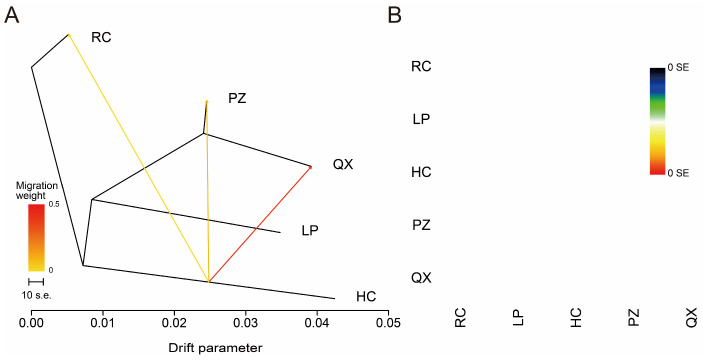
TreeMix analysis of five pig breeds. (**A**) Phylogenetic trees of five breeds showing three migration events. The branch length indicates the degree of population differentiation, and the arrow direction indicates the direction of gene flow from the donor to the recipient population. Warmer colors indicate stronger gene flow. Scale bars represent 10 times the standard error for the mean (S.E.) of the estimated entries in the sample covariance matrix. (**B**) Residual plot under three migration events expressed as the number of standard errors of deviation. Red indicates that the observed genetic similarity between two groups is higher than the model’s predicted value, while blue indicates the opposite. White color indicates good agreement between predicted and observed data.

In the TreeMix analysis, when the number of migration events was three, the color of all blocks in the residual graph was white, indicating that the simulation result is the most accurate (Figure 4B). Therefore, we set the number of migration events to three and identified the events that occurred in the five breeds. Gene flow occurred from HC to RC, PZ, and QX, with a higher degree of gene exchange from HC to QX (Figure 4A).

### 3.4. Analysis of Genetic Relationships

**Figure 5:**
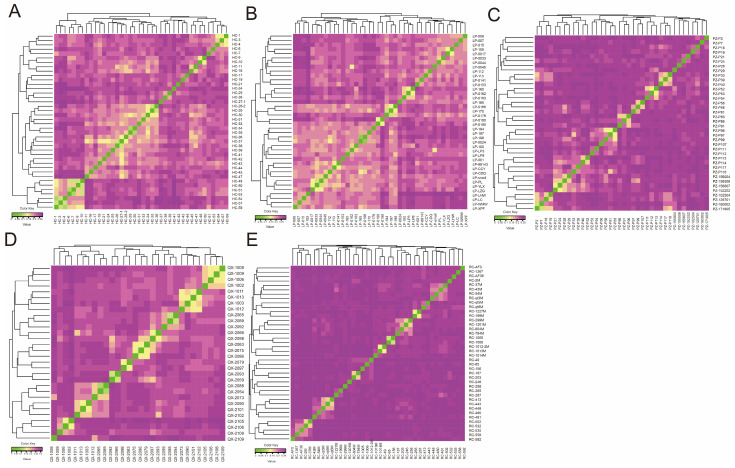
(**A**–**E**) Identity-by-state (IBS) distance matrixes for HC, LP, PZ, QX, and RC. Each square represents the genetic distance between two individuals. Values are positively associated with genetic distance. HC, Hechuan Black pigs; LP, Luopanshan pigs; PZ, Penzhou Mountain pigs; QX, Quxi pigs; RC, Rongchang pigs.

The IBS distances in HC, LP, PZ, QX, and RC were 0.217 ± 0.028, 0.226 ± 0.026, 0.239 ± 0.023, 0.259 ± 0.032, and 0.246 ± 0.016, respectively, indicating significant genetic distances between individuals within each population (Figure 5). Some individuals in the HC, LP, PZ, and QX populations had similar genetic distances, indicating a risk of inbreeding and underscoring the need to improve breeding programs.

**Figure 6:**
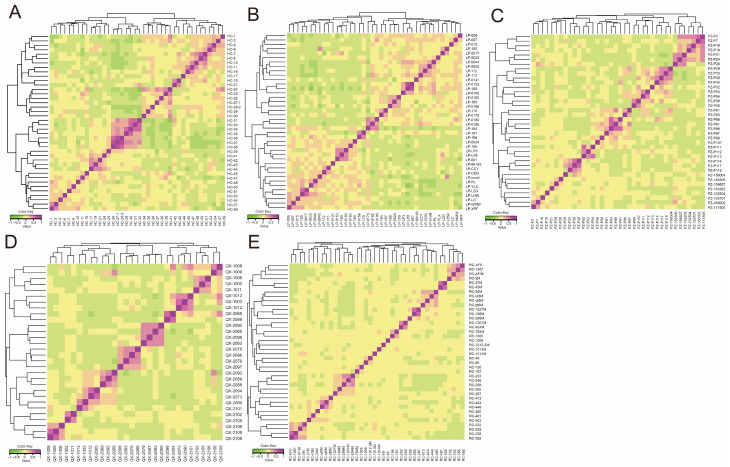
(**A**–**E**) G-matrix heat maps of HC, LP, PZ, QX, and RC. Each square represents the relatedness between two individuals. Values are positively associated with relatedness. HC, Hechuan Black pigs; LP, Luopanshan pigs; PZ, Penzhou Mountain pigs; QX, Quxi pigs; RC, Rongchang pigs.

The results of the phylogenetic analysis using the G-matrix showed that the phylogenetic relationship coefficients for HC, LP, PZ, QX, and RC were 0.0432 ± 0.0929, 0.0416 ± 0.0762, 0.0326 ± 0.0874, 0.0375 ± 0.1060, and 0.0163 ± 0.0616, respectively (Figure 6). Most individuals in the HC, LP, PZ, and QX populations had moderate genetic relatedness; however, a few had closer relatedness, indicating a higher risk of inbreeding. RC had fewer individuals with close genetic relatedness.

## 4. Discussion

Chinese domestic pig breeds have distinct genetic traits shaped by natural selection as well as environmental, cultural, and historical factors. These genetic traits increase the diversity of the gene pool. Recent African swine fever outbreaks have significantly challenged conservation efforts for pig breeds in China, resulting in a decline in pig populations and genetic diversity. This study analyzed the genetic diversity and population structure of five pig breeds in Chongqing using SNP chips, clarifying the genetic uniqueness and diversity of local pigs, providing a basis for identifying the risk of inbreeding and for monitoring genetic diversity.

The genetic diversity of a population is influenced by natural selection, genetic drift, gene flow, human activities, climate change, and other factors. A study investigated the genetic diversity of 56 pig breeds from China and found that Ho values ranged from 0.44 to 0.87 [34]. We found that the Ho values of Chongqing pig breeds were relatively low, ranging from 0.2958 to 0.3624, which is consistent with other studies showing that the Ho values of Licha Black, Fengjing, and Tongcheng pigs were also low (0.351, 0.301, and 0.320) [21,22,35]. In this study, Ho values in all pig breeds were higher than He values. Conversely, a previous study showed that Ho values in PZ and RC were lower than He values [36]. These results indicate that the Ho of Chinese indigenous pig breeds has declined in recent years, with Ho > He becoming more common [22,23,37]. In addition, the Ne values of Chongqing pig breeds were below 5, which is lower than those of other Chinese breeds, such as Licha Black pigs (8.7) and Jiangquhai pigs (195), as well as breeds from Italy, Germany, and France [35,38,39,40]. These results suggest that Chongqing pig populations may face risks such as reduced genetic diversity and inbreeding depression. Thus, it is necessary to formulate effective selection and mating plans and to introduce new bloodlines to enhance the reproductive efficiency and population size of local pig populations.

LD analysis can reveal the degree of selection and genetic polymorphism in populations over time. LD decay decreases as the degree of selection increases. The analysis of LD decay reveals differences in the degree of selection among subpopulations [41]. We found that RC had the slowest LD decay, indicating weak selection, whereas HC had the fastest LD decay, indicating strong selection. FST values of 0–0.05, 0.05–0.15, and >0.15 indicate low, moderate, and high genetic differentiation, respectively. The FST values for the five pig breeds ranged from 0.0748 to 0.1958, which is similar to the values for other pig breeds (0.06–0.237). Genetic differences among pig breeds are moderate to high [42]. All FST values between HC and the other four breeds were above 0.15 (0.1556–0.1958), indicating a high level of genetic differentiation. One explanation is that HC conservation farms use closed breeding, which affects population diversity through selective breeding [43]. Differences in specific genes among pig breeds reflect intraspecific genetic diversity and are the leading cause of breed formation and trait variation [44]. HC may have more unique genes, including those related to stress resistance and meat quality, than the other four breeds, making HC valuable for breeding.

The results of PCA and the evolutionary tree showed that the five groups were completely independent and separated, indicating significant genetic differentiation among them. In addition, the analysis of population structure showed that there are varying degrees of gene flow among the breeds, indicating that several breeds have undergone gene exchange. For instance, in the HC group, there is introgression from RC, LP, and PZ, while in the RC group, there is a small amount of introgression from HC and LP. Admixture analysis based on allele frequencies can estimate ancestry [45]. TreeMix analysis constructs a maximum likelihood phylogenetic tree based on the covariance of population allele frequencies. This analysis can accurately assess recent and high-intensity gene flows but not ancient or weak gene flows [33]. The TreeMix results revealed the complex evolutionary relationships among pig breeds. There was relatively weak gene flow from HC to RC, and no gene flow from RC to HC. This result indicates that gene flow from RC to HC occurred long ago, which may be related to the historical commercial use of RC. The conservation work for Rongchang pigs started early, but most conservation farms have implemented closed management systems, thereby blocking genetic exchange with external populations [20]. However, due to the geographical location, the main regions of production of RC and HC are relatively close to each other, allowing gene flow from HC to RC. The results also revealed a small gene flow from HC to PZ. A possible reason is that PZ samples were collected at a conservation farm, which was formerly a company engaged in Black pig hybridization. This company introduced HC for hybridization breeding work. There was high gene flow from HC to QX. A possible reason is that the conservation work for QX has only started in the past 2 years. Before that, local farmers practiced their own breeding; in addition, they distinguished varieties only by color and had no awareness of the differences between them. HC were more favorable to farmers than LP and PZ due to its larger size. The increased convenience of transportation in recent years has increased gene flow from HC to QX. There was no gene flow from HC to LP. We speculate that this is because although the ancestral groups of HC and LP had gene exchange, subsequent reproductive isolation or the selective elimination of non-adaptive genes led to low detection rates.

The conservation of Chinese pig breeds involves closed breeding. Many breeds, including Fengjing pigs and Jianhebaixiang pigs, have a high risk of inbreeding due to closed breeding, which poses a significant challenge to breed conservation [17,21]. The results showed that the IBS distances between individuals of the five pig breeds were large, indicating that these breeds have significant genetic differences and unique genetic characteristics due to long-term evolution and selection. Some individuals had similar IBS distances, indicating a higher risk of inbreeding. G-matrix analysis supports this conclusion, as some animals were more closely related than expected, indicating inbreeding within the population. Long-term inbreeding may reduce reproductive performance, disease resistance, and other traits. Therefore, new breeds should be introduced into conservation programs to improve population structure and prevent the loss of valuable genetic resources due to inbreeding depression, ensuring the sustainable conservation and use of local pig breeds in Chongqing.

This study has limitations. First, the sample size was small; thus, studies with larger sample sizes are necessary to validate the results. Second, the SNP chips used in this study were developed based on international breeds (Large White and Landrace) and were not targeted at Chongqing breeds, thus failing to cover the entire genome. Thus, unique or rare variants were not identified, potentially decreasing genetic diversity. We will conduct an in-depth analysis of the genetic mechanisms underlying the adaptive evolution of Chongqing pigs, providing a theoretical basis for the protection, improvement, and sustainable utilization of local pig breeds.

## 5. Conclusions

The results show that the pig breeds from Chongqing have relatively high levels of genetic diversity. Each of the five pig breeds from Chongqing has independent evolutionary trajectories, with varying degrees of gene infiltration among the breeds. There was a strong gene flow from HC to QX. These results will help improve our understanding of the genetic characteristics of Chongqing pigs and their potential applications, providing a basis for formulating science-based and effective protection strategies. Further research on the selected signals during the adaptive evolution of each breed is needed to analyze the genetic basis of disease and stress resistance traits and to identify molecular markers for the development and commercial utilization of local pigs in Chongqing.

## Figures and Tables

**Table 1:** Pig breeds from Chongqing, China.

Breed	Number of Individuals	Location
HC	40	Hechuan
LP	40	Tongnan
PZ	38	Fulin
QX	30	Fengdu
RC	40	Rongchang

HC, Hechuan Black pigs; LP, Luopanshan pigs; PZ, Penzhou Mountain pigs; QX, Quxi pigs; RC, Rongchang pigs.

**Table 2:** Genetic diversity parameters of five pig breeds from Chongqing, China.

Breed	MAF	He	Ho	Ne	PN	Pi
HC	0.135	0.2751	0.2958	2.9	0.5090	0.2785
LP	0.150	0.2884	0.3144	4.1	0.5644	0.2921
PZ	0.173	0.2948	0.3084	3.5	0.7002	0.2987
QX	0.229	0.3302	0.3624	2.7	0.8946	0.3359
RC	0.153	0.3012	0.3044	4.6	0.5744	0.305

HC, Hechuan Black pigs; LP, Luopanshan pigs; PZ, Penzhou Mountain pigs; QX, Quxi pigs; RC, Rongchang pigs. MAF, minor allele frequency; He, expected heterozygosity; Ho, observed heterozygosity; Ne, effective population size; PN, polymorphic marker ratio; Pi, nucleotide diversity.

## Data Availability

The original contributions presented in this study are included in the article. Further inquiries can be directed to the corresponding author.
